# Neurological manifestations in patients with antiphospholipid syndrome

**Published:** 2013

**Authors:** Masoud Etemadifar, Leila Dehghani, Soheil Tahani, Nafiseh Toghianifar, Marzieh Rahaimi, Nahid Eskandari

**Affiliations:** 1 Professor, Isfahan Multiple Sclerosis and Neuroimmunology Research Center, Isfahan Eye Research Center, Ophthalmology Ward, Feiz Hospital, Isfahan University of Medical Sciences, Isfahan, Iran; 2 PhD Student, Department of Medical Sciences, School of Medicine, Najafabad Branch, Islamic Azad University, Isfahan, Iran; 3 Department of Medical Sciences, School of Medicine, Najafabad Branch, Islamic Azad University, Isfahan, Iran; 4 Assistant Professor, Department of Neurology, School of Medicine, Isfahan University of Medical Sciences, Isfahan, Iran; 5 Assistant professor, Department of Immunology, School of Medicine, Isfahan University of Medical Sciences, Isfahan, Iran

**Keywords:** Antiphospholipid Syndrome, Miscarriage, Neurological Manifestation

## Abstract

**Background:**

Anti-phospholipids syndrome (APS) is considered a non inflammatory auto-immune disease with a significant thrombophilic risk with varied clinical manifestations. The purpose of the current study was to investigate the frequency of thrombotic and non-thrombotic events in patients with APS.

**Methods:**

In this retrospective study, 102 definite APS subjects were recruited (2007-2011) at Alzahra Hospital, Isfahan, Iran. The patients were referred to Multiple Sclerosis Clinic with the diagnosis of definite APS according to 2006 Sydney's criteria. Disorders associated with APS such as pregnancy complication, vascular thrombosis and livedo reticularis (LR) were assessed. Neurological signs and symptoms such as cognitive dysfunction were recorded. Data analyses were performed using SPSS software and P < 0.05 were considered to be statistically significant.

**Results:**

Our findings showed that majority of female gender, higher rate of ischemic thrombotic stroke and high miscarriage lied in a large number of APS patients.

**Conclusion:**

Overall recurrent miscarriage is a common complication among (antiphospholidpid antibody) aPL patients. Furthermore, ischemic stroke is the second common neurological manifestations of APS patients.

## Introduction

Anti-phospholipids syndrome (APS) is considered a non inflammatory auto-immune disease. It is associated with a significant thrombophilic risk with varied clinical manifestations that usually mimics other syndrome. This syndrome includes a variety of clinical presentations such as vascular thrombosis and spontaneous abortions, preterm delivery due to eclampsia or severe preeclampsia, and several laboratory abnormalities, detected at least twice, 12 weeks apart (anticardiolipin, anti-β_2_-glycoprotein I or lupus anticoagulant) together with at least one laboratory criterion.^1^ The arterial and venous thrombosis that is commonly associated with APS reflects hypercoagulability. The most prevalent APS-related clinical manifestation are deep vein thrombosis (DVT) of the lower extremities, pulmonary thromboembolism (PTE), ischemic stroke (IS) and amaurosis fugax with or without arterial occlusion of the retina vessels.^2, 3^ Cerebrovascular accidents (CVA) either stroke or transient ischaemic attacks (TIA) are the most typical arterial thrombotic manifestations. The most common non-thrombotic features are thrombocytopenia, cardiac vascular abnormalities, microangiopathic nephropathy, and livedo reticularis (LR). Chorea, seizures, and migraine such as headache and transverse myelitis as other neurologic disorder of APS have also been reported.^4^ The purpose of the current study was to investigate the frequency of thrombotic and non-thrombotic events in patients with APS.

## Materials and Methods

Based on retrospective study, we reviewed on 102 patients who were referred to Multiple Sclerosis Clinic with the diagnosis of definite APS made by a neurologist according to 2006 Sydney's criteria at Alzahra Hospital, Isfahan, Iran (2007-2011). In this study, age, sex, family and medical history, general neurologic and rheumatologic symptoms and physical examination in all the patients were recorded. Additionally, disorders associated with APS such as complication of pregnancy, thrombosis and LR were assessed. We analyzed all the clinical, laboratory, brain magnetic resonance (MR) and abnormalities findings of patients with APS in an attempt to identify parameters.

### Statistical Analyses

Data analysis was performed using SPSS for Windows 18.0 (SPSS Inc., Chicago, IL, USA). Results showed as mean ± standard deviation (SD) or percentages as appropriate. Chi-square, Fisher's exact test, and Spearman correlation coefficient were used for comparisons between neurological and non-neurological complications, when applicable. Logistic regression test was also used to analyze associations between variables. P < 0.05 was considered to be statistically significant.

## Results

The mean age of the patients was 35.7 ± 11.4 (women) and 43.8 ± 14.2 (men) years, respectively. Most of the women were married (59.58%, n = 53) and more than half (59.95%) of them had history of miscarriage. Among of these women, 25.3% had deep vein thrombosis (DVT). Of all types of vascular thrombosis, our study showed that DVT was the most prevalent presentation (28.42%).

48.41% of the patient with neurological disorders had a history of stroke which 37.89% of whom had ischemic stroke and 10.52% had TIA. Moreover, 38.88% (n = 14) of the patients with IS, had stroke in territory of anterior circulation and 19.44% of stroke was (n = 7) in posterior circulation. Unilateral optic neuritis had occurred in 11 patients. DVT was also observed in patients with cerebral disorder (58.8%), simultaneously. LR was positive in %14.56 (n = 15) of the patients and it noticed at a mean age of 27 years. Present results showed that there was a statistically significant association between miscarriage and cerebral manifestations. In addition, Magnetic resonance imaging (MRI) showed evidence of ischemic lesions (51.45%). More neurological disorders results showed in the Table 1.

**Table 1:** Disorders associated with antiphospholipid syndrome

	Number	Percentage
Gender ratio, Female/Male (%F)	90/13	87.37/12.62
Age (years) (mean ± SD)	36.3 ± 11.4 F (35.7 ± 11.4) M (43.8 ± 14.2)	
Pregnancy complications		
Miscarriage	53	59.55
Preeclampsia	3	3.37
Vascular Thrombosis		
DVT	27	28.42
PTE	7	7.36
CVT	4	3.92
Neurological Problems		
Headache	38	13.72
Ischemic Stroke(infarct)	36	37.89
Seizure	20	19.60
ON	11	10.78
TIA	10	10.52
Transverse myelitis	2	
Peripheral neuropathy	1	
Depression	11	10.67
Dementia	2	1.94
Livedo reticularis	15	14.56
MRI abnormality	53	51.45

## Discussion

The antiphospholipid syndrome is described as a disease of our time, classified as an auto-immune condition antibody mediated and with clinical links in some cases to other autoimmune conditions (lupus, Sjo gren's Syndrome and Hashimoto's thyroiditis). It is also almost certainly genetically mediated with many positive family cohorts described. The pathogenesis of the thrombosis is still discussed, with direct action by antiphospholipid antibody (aPL) on platelet membranes, on clotting proteins and on the endothelium all being described.APS estimated to be responsible for 10% of all deaths in many non-industrial countries.^5, 6^

Our study demonstrated that predominance of female gender, high rate of ischemic thrombotic stroke which the first explanation (aPL) would increase the thrombophilic risk, and a second is required so that the clotting takes place. Moreover, in consist with our results, a recent multicenter prospective analysis of 1,000 patients with APS has shown that thrombotic complications are the most common cause of death in APS patients.^6^

In addition, this study demonstrated high rate of miscarriage was positioned in a large number of APC. Research has shown that recurrent miscarriage is a common complication among aPL patients. Antiphospholipid antibodies are detected in more than 10% of patients with recurrent miscarriage (RM),^7^ and they have emerged as one of the most common causes of this reproductive complication.^5, 7^ Therefore, the most frequent acquired thrombophiliain RM patients is APS. In this order, a study^8^ on 500 women with history of at least three consecutive miscarriages demonstrated no difference in the gestation of miscarriages between aPA positive and APA negative women, and rate of aPA positive with recurrent miscarriage were varies (11-42%). Our study indicated more than half percentage of patients had a history of miscarriage and 35% of patients with aPL had a history of at least three abortions. Some studies have shown that the pair of small vessel thrombosis introduced as a major cause of fetal loss.^9^ The present study confirmed that 25.35% of patients with miscarriage had DVT concurrently and also found global correlation between miscarriage and cerebral manifestations.

Of other neurological symptoms, ischemic stroke is the second common neurological manifestations of APS. This study identified this issue and showed that more than half of patients had ischemic stroke (IS) and DVT. The previous reported prevalence of optic neuritis (ON) in patients with APL have varied from 5-75%.^10^ Our results showed the frequency of ON was 10.78%. Several cognitive problems can be implicated in this syndrome ranging from simple cognitive dysfunction to severe dementia.^10^ Hence, depression and dementia were found in some patients of the present study which indicated an association between these disorders and APS. Therefore, APS may be explored as possible risk factors for depression or dementia disorders.

Many studies introduced LR as a frequent skin vasculopathy in patients with APS which resulted from cerebrovascular disorders. In this study, the rate of stroke in patients with LR in the absence of other vascular risk factors was high.

MRI differences between MS and primary antiphospholipid antibody syndrome (PAPLS) patients and MRI of the patients showed evidence of ischemic lesions. This indicated that the presence of APLA in MS was subject to dynamic changes over time that leads to their higher prevalence with ongoing disease duration. Longitudinal studies on larger patients with various disease subtypes are needed to confirm MRI findings and to better define the association between aPL and tissue damage as reflected by MRI changes.

## Conclusion

In APS patients, recurrent miscarriage and ischemic thrombotic stroke are common complications and it could be well thought-out as an important issue.
